# Association between epicardial adipose tissue and adverse outcomes in coronary heart disease patients with percutaneous coronary intervention

**DOI:** 10.1042/BSR20182278

**Published:** 2019-05-07

**Authors:** Changqing Lu, Helei Jia, Zhentao Wang

**Affiliations:** 1 Department of Emergency, The Second Affiliated Hospital of Henan University of Chinese Medicine (Henan Province Hospital of Traditional Chinese Medicine), Zhengzhou, Henan Province 450002, China; 2 Department of Cardiovascular, Henan Province Hospital of Traditional Chinese Medicine, Zhengzhou, Henan Province 450002, China

**Keywords:** Clinical prognosis, Coronary heart disease, Epicardial adipose tissue, Major adverse cardiovascular events, Percutaneous coronary intervention

## Abstract

We assessed the relationship between the volume of epicardial adipose tissue and long-term outcomes in patients with coronary heart disease (CHD) undergoing percutaneous coronary intervention (PCI). The patients with CHD were followed for at least 2 years after PCI. The epicardial adipose tissue volume (EATV) was measured using multi-slice computed tomography. Cox regression analysis was used to examine the relationship between EATV and clinical outcome. In this study, 500 patients were enrolled and followed up for a median of 25.2 months. The incidence of adverse cardiovascular events was 12.4%. No significant differences were observed in age, sex, proportion of patients with hypertension or diabetes, smoking, drinking, total cholesterol, triglyceride, high-density lipoprotein, or unstable angina pectoris among different EATV quartiles (*P*>0.05). The EATV was associated with body mass index (*P*<0.0001), low-density lipoprotein level (*P*=0.039), high-sensitivity C-reactive protein level (*P*<0.001), uric acid level (*P*=0.004), adiponectin level (*P*<0.001), and left ventricular ejection fraction (*P*<0.001). Kaplan–Meier analysis indicated a significant difference in survival rate of patients in EATV quartile 1 versus 4 (*P*=0.019). After adjusting for confounding factors, EATV quartile 4 (>216.15 cm^3^) was still associated with adverse cardiovascular outcomes (HR = 1.98, 95% CI: 1.15–4.47, *P*=0.023) compared with quartile 1 (<101.58 cm^3^). Our data suggest that EATV is an independent predictor of long-term major adverse cardiovascular events in CHD patients after PCI. Therefore, assessment of EATV using multi-slice computed tomography may contribute to risk stratification in these patients.

## Introduction

Coronary artery disease (CAD) involves a reduction in blood flow to heart muscles due to a buildup of plaque in the coronary arteries. It is the most common cardiovascular disease [1]. Percutaneous coronary intervention (PCI) has become an important method for diagnosing and treating coronary heart disease (CHD). PCI can effectively realize coronary reperfusion and relieve ischemia symptoms with slight trauma and less pain, while significantly reducing the hospitalization rate and mortality and improving the quality of life of patients [2]. However, patients still have some postoperative complications, such as stent restenosis and stent intimal hyperplasia [3], which increase the risk of major adverse cardiovascular events (MACEs). Although the postoperative incidence of intravascular thrombosis has decreased significantly with the adoption of drug-coated stents and antiplatelet drugs, postoperative restenosis can still occur in some patients [4]. Therefore, it is important to identify predictors of complications after PCI.

Epicardial adipose tissue (EAT) is visceral adipose tissue between the myocardium and pericardium, covering three quarters of the heart surface. Like other adipose tissues, EAT can lead to the formation of non-calcified plaques in the coronary arteries by secreting a variety of cytokines and inflammatory factors. Perivascular adipose cytokines accumulate in EAT, which leads to a series of clinical cardiovascular events. The amount of EAT is reported to be associated with adverse cardiac structure and function and atrial fibrillation (AF) severity EAT accumulation also appears to be associated with stroke and MACEs in AF [5,6]. An increase in EAT was strongly correlated with the incidence and duration of AF and might be an independent risk factor for AF [7]. Pierdomenico et al. suggested that the epicardial adipose tissue volume (EATV) was independently related to left ventricular hypertrophy after adjusting for certain confounding factors, including hypertension [8]. Therefore, we hypothesized that EAT contributes to pathological cardiac remodeling and may be associated with the clinical outcome of CHD patients. Therefore, we conducted a retrospective study to examine MACEs occurring within 3 years after PCI in patients with CHD, and to determine the relationship between EATV and the long-term prognosis of these patients.

## Materials and methods

### Study population

We enrolled CHD patients who underwent PCI from August 2013 to July 2017 in the Emergency Department of The Second Affiliated Hospital of Henan University of Chinese Medicine. Clinical data and outcomes were collected. The inclusion criteria were age 18 to <75 years, diagnosis of CHD confirmed by coronary angiography, underwent PCI with available clinical and outcome data after treatment, acute coronary syndrome, and ST elevation and non-ST elevation myocardial infarction. Criteria for exclusion were severe infection, cardiogenic shock, congenital heart disease, valvular disease, primary pericardial disease or cardiomyopathy, renal dysfunction (glomerular filtration rate < 60 ml/min/1.73 m^2^), history of myocardial infarction, revascularization, previous PCI or coronary artery bypass grafting, thrombocytopenia, severe peptic ulcer, and bleeding disorder.

The present study was approved by the ethics committee of The Second Affiliated Hospital of Henan University of Chinese Medicine. The research was conducted in accordance with the World Medical Association Declaration of Helsinki, and all subjects provided informed consent.

### Data collection

General demographic data, including age, sex, history of smoking (at least five cigarettes a day for more than 1 year), and history of disease, were collected using a standard questionnaire. Body mass index (BMI) was calculated as weight/height^2^ (kg/m^2^) and classified as normal (18.5 ≤ BMI < 24), overweight (24 ≤ BMI < 28), and obese (BMI ≥ 28) [9]. Hypertension was diagnosed if one of the following was present: systolic blood pressure ≥ 140 mmHg, diastolic blood pressure ≥ 90 mmHg, or taking an antihypertensive drug before admission. Diabetes was defined as a fasting blood glucose level ≥ 7.8 mmol/l or 2-h postprandial glucose level ≥ 11.1 mmol/l. Venous blood was collected from fasting patients in the morning within 24 h after admission. The serum levels of low-density lipoprotein cholesterol (LDL-C), high-density lipoprotein cholesterol (HDL-C), triglycerides (TG), total cholesterol (TC), fasting glucose, high-sensitivity C-reactive protein (hs-CRP), and adiponectin were determined by a fully automatic biochemical analyzer. Dyslipidemia was defined as a TC level > 5.18 mmol/l, TG level > 1.7 mmol/l, LDL-C level > 3.37 mmol/l, or HDL-C level < 1.04 mmol/l.

### EATV examination

The EATV of all patients was measured using 64-slice spiral computed tomography (CT) before PCI. The operator outlined the boundary of the heart in the transverse, sagittal, and coronary views using the volume measurement tools of the GE AW 4.6 software work station. The cardiac volume was determined by manually outlining the wall of the pericardium, with a 6-mm space between two lines. After outlining the heart, the operator marked the color of adipose tissue by adjusting the CT value from −250 to −50 HU. The volume was calculated in accordance with the range of CT values. All procedures were performed by one experienced technician and confirmed and checked by the senior technician.

### Percutaneous coronary intervention

The indications for PCI in acute coronary syndrome were as follows: (1) acute myocardial infarction or newly developed left bundle branch block (LBBB) and able to undergo PCI within 12 h after symptom onset, (2) severe congestive heart failure or pulmonary edema within 12 h of symptom onset, (3) severe congestive heart failure, hemodynamic or electrocardiogram (ECG) instability, or evidence of persistent ischemia within 12–24 h of symptom onset, and (4) unsuitable for intravenous thrombolysis, with symptoms for less than 12–24 h, and severe congestive heart failure or hemodynamic or ECG instability/persistence. Remedial PCI was conducted after thrombolysis failure for patients with ST segment elevation or newly found LBBB myocardial infarction, myocardial-infarction-induced cardiogenic shock for less than 36 h, and under 75 years of age; severe congestive heart failure or pulmonary edema with symptoms for less than 12 h; or evidence of hemodynamic or electrical instability or persistent myocardial ischemia. If the patient had recurrent angina with no objective evidence of myocardial ischemia or myocardial infarction, PCI was determined by weighing the available evidence. PCI was performed for stable CHD when the medical treatment was unsatisfactory.

All patients underwent coronary angiography before PCI, including the left anterior descending branch, left circumflex artery, and right coronary artery. Positive findings were as follows: arterial stenosis of at least one primary branch or ≥ 50% of a single branch lesion; arterial stenosis ≥ 50% of two primary branches; and arterial stenosis ≥50% of all three branches or the left primary branch with three lesions [10]. Patients were given 300-mg aspirin and 300-mg clopidogrel before PCI and continued to take 100-mg aspirin and 75-mg clopidogrel daily after PCI. During the procedure, the patients were given low-molecular-weight heparin (100 IU/kg). The patients were given statins, β-blockers, angiotensin-converting enzyme inhibitors, or calcium antagonists for prevention according to disease progression.

### Outcomes

The primary outcomes were MACEs, including death, myocardial infarction, and revascularization. For repeat revascularization of the target lesion, revascularization followed restenosis or stent thrombosis within 5 mm of the stent at both ends (edge effect). For revascularization of non-target lesions, revascularization occurred in non-target coronary artery lesions after PCI, including non-stented sites in the target vessels and non-target vessels [11]. In this study, non-target lesion revascularization did not include those patients who underwent multi-stage PCI treatment, which was considered a completed treatment. Myocardial infarction was defined as elevation of CK-MB or troponin-I more than 2-fold the normal levels, with persistent chest pain for more than 20 min or typical ECG changes (Q wave or LBBB or dynamic ST-T wave changes). The follow-up was conducted by telephone and in the outpatient clinic.

### Statistical analysis

All analyses were performed on the SPSS 20.0 station. EATV was divided into four groups according to quartiles: <25, 25–50, 50–75, and >75%. Quantitative data were expressed as means ± standard deviation. One-way analysis of variance was used to compare three or more groups. Non-normally distributed quantitative data were expressed as medians and interquartile ranges and compared using the non-parametric Kruskal–Wallis test. Qualitative data were expressed as the count and percentage, and the χ² test was used. Kaplan–Meier analysis was used to compare the survival curves among different EATV quartiles (pairwise comparisons were also made using this analysis). We used univariate and multivariate Cox regression analyses to assess the relationships between EAT and MACEs after adjusting for potential confounding factors, including age, sex, smoking, drinking, hypertension, diabetes mellitus, BMI, TC, TG, LDL-C, HDL-C, hs-CRP, creatinine, uric acid, vessel lesions, type of CAD (acute coronary syndrome vs stable CHD), and the left ventricular ejection fraction (LVEF). A restricted cubic spline plot was generated to reveal the relationships between EATV and the hazard ratios of various adverse outcomes (the reference value of EATV was 42.8 with five knots Q1, Q25, Q50, Q75). *P*<0.05 was considered to indicate significance.

## Results

### Baseline characteristics of the study population

A total of 500 consecutive patients with CHD were referred to our center for PCI. The patients comprised 296 males (59.2%) and 204 females (40.8%), with a mean age of 58.9 years. The median follow-up time was 25.2 months (interquartile range, 13.2–40.1). Of the patients, 30.2% (*n*=151) had diabetes mellitus, 40.8% (*n*=204) hypertension, 48.2% (*n*=241) a history of smoking, 59.8% (*n*=299) a history of drinking, 37.2% (*n*=188) acute myocardial infarction, and 44.2% (*n*=221) unstable angina pectoris. In our study cohort, 18.6% (*n*=93) of the patients had a single-vessel lesion and 81.4% (*n*=407) multi-vessel lesions.

The study population was divided into four groups according to EATV quartile: <101.58, 101.58–156.15, 156.15–216.15, and >216.15 cm^3^. Table 1 compares the clinical data of the patients among the EATV quartiles. There were no significance differences in age (*P*=0.188), sex (*P*=0.899), rate of hypertension (*P*=0.413) or diabetes (*P*=0.843), or history of smoking (*P*=0.855) or drinking (*P*=0.066). A significant difference in BMI was observed among the four quartiles (*P*<0.0001). No significant differences were observed in the levels of blood glucose (*P*=0.080), TC (*P*=0.750), TG (*P*=0.245), or HDL-C (*P*=0.235). The LDL-C level was higher in quartile 4 than quartiles 1 and 2 (*P*=0.039). Compared with quartile 1, quartile 4 had higher levels of hs-CRP (*P*<0.0001), creatinine (*P*<0.0001), uric acid (*P*=0.004), and adiponectin (*P*<0.0001). The rates of acute myocardial infarction and unstable angina pectoris were roughly equal among all four groups (*P*=0.798). The LVEF was inversely associated with the EATV, decreasing with increasing EATV (*P*<0.001).

**Table 1:** Baseline demographics of study population by EATV

Factors	Quartile 1 (*n*=118)	Quartile 2 (*n*=138)	Quartile 3 (*n*=120)	Quartile 4 (*n*=124)	F/χ^2^/U	*P*-value
Age (year)	58.3 ± 9.5	57.8 ± 10.4	59.6 ± 9.8	60.1 ± 8.7	1.601	0.188
Male (*n*, %)	65( 55.1%)	82 (59.4%)	72 (60.0%)	77 (62.1%)	0.212	0.899
Hypertension (*n*, %)	43 (36.4%)	44 (31.9%)	40 (30.0%)	31 (25.0%)	3.950	0.413
Diabetes (*n*, %)	37 (31.4%)	41 (29.7%)	40 (33.3%)	33 (26.6%)	1.406	0.843
Smoking (*n*, %)	54 (45.8%)	62 (44.9%)	60 (50.0%)	63 (50.8%)	1.335	0.855
Drinking (*n*, %)	58 (49.2%)	92 (66.7%)	71 (59.2%)	78 (62.9%)	8.788	0.066
BMI, kg/m^2^	24.5 ± 3.2	24.6 ± 2.8	26.4 ± 3.1	28.4 ± 3.5	42.596	<0.0001
Blood glucose, mmol/l	6.4 ± 2.1	5.9 ± 0.9	6.1 ± 1.6	6.2 ± 1.5	2.266	0.080
TC, mmol/l	4.6 ± 1.8	4.5 ± 0.9	4.6 ± 1.1	4.7 ± 1.1	0.404	0.750
TG, mmol/l	1.7 ± 1.4	1.6 ± 0.8	1.9 ± 1.5	1.8 ± 1.2	1.389	0.245
LDL-C, mmol/l	2.7 ± 0.8	2.8 ± 0.8	2.9 ± 0.9	3.0 ± 0.9	2.808	0.039
HDL-C, mmol/l	1.1 ± 0.2	1.1 ± 0.2	1.0 ± 0.2	1.0 ± 0.2	1.492	0.235
Hs-CRP, mg/l	5.4 ± 0.8	5.2 ± 0.5	7.6 ± 0.7	9.8 ± 1.6	604.647	<0.0001
Creatinine, mmol/l	65 (62–75)	70 (61–78)	77 (60–83)	80 (69.5–98)	15.75	<0.0001
Uric acid, mmol/l	335.4 ± 78.5	333.8 ± 99.8	359.7 ± 92.3	368.4 ± 89.4	4.583	0.004
Adiponectin, mg/l	12.1 ± 3.5	11.6 ± 3.9	8.9 ± 4.2	8.1 ± 5.3	26.357	<0.0001
AMI (*n*, %)	40 (33.9%)	40 (29.0%)	52 (43.3%)	54 (43.5%)	8.608	0.072
UAP (*n*, %)	42 (35.6%)	69 (50.0%)	55 (45.8%)	55 (44.4%)	5.557	0.235
Single vessel (*n*, %)	30 (25.4%)	20 (14.5%)	24 (20.0%)	19 (15.3)	6.201	0.184
Multiple vessel (*n*, %)	97 (82.2%)	115 (83.3%)	93 (77.5%)	102 (82.3%)	1.657	0.798
LVEF (%)	59.6 ± 6.8	58.1 ± 7.1	56.1 ± 6.3	52.3 ± 9.6	21.519	<0.0001

**Abbreviations**: AMI, acute myocardial infarction; BMI, body mass index; HDL, high density lipoprotein; Hs-CRP, high-sensitivity C-reactive protein; LDL, low density lipoprotein; LEVF, left ventricular ejection fraction; TC, total cholesterol; TG, triglyceride; UAP, unstable angina pectoris.

### Follow-up outcomes

The incidence of MACEs was 12.4% (*n*=62), which included 5 deaths, 25 myocardial infarctions, and 32 revascularization cases. The incidences of MACEs in the four quartiles were 5.9% (*n*=7), 10.9% (*n*=15), 13.3% (*n*=16), and 19.4% (*n*=24), respectively, and differed significantly among the four groups (*P*=0.033) that in quartile 4 was significantly higher than that in quartile 1 (*P*_adjusted_= 0.0017, *α* = 0.008). There were no differences between any other two quartiles (*P*>0.05).

### Outcomes according to EATV

**Figure 1:**
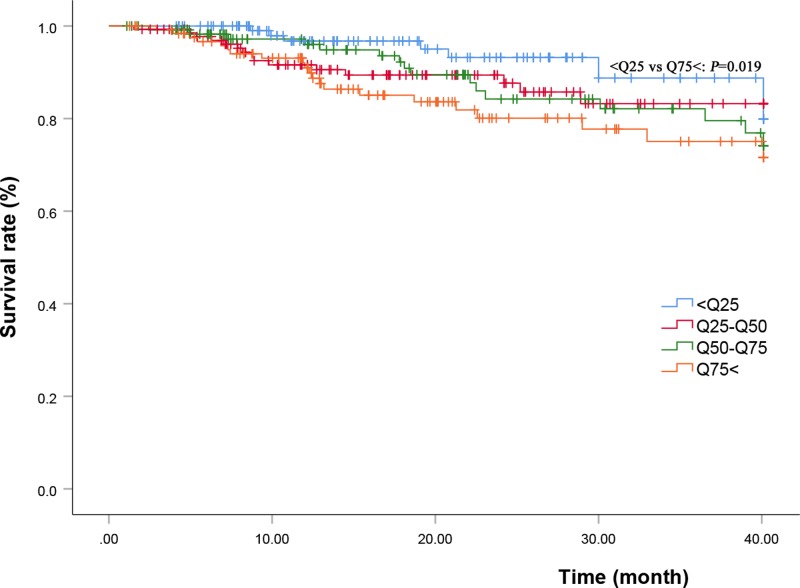
Overall survival rate of different levels of EAT

**Figure 2:**
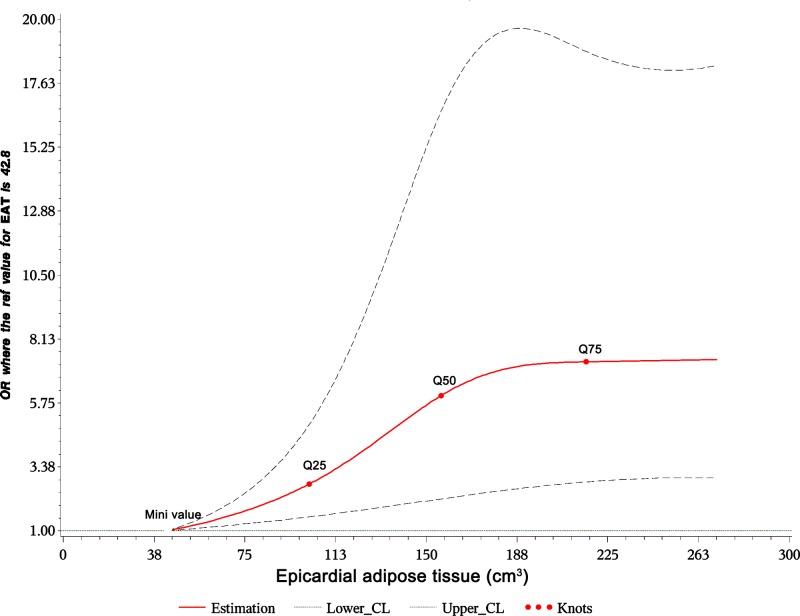
Restrictive cubic spline plots between EAT and MAC hazard ratio

Table 2 shows the results of univariate Cox regression analyses. Age ≥ 60 years (HR = 2.32, 95% CI: 1.87–6.15), obesity (HR = 1.84, 95% CI: 1.35–2.49), hs-CRP (HR = 1.39, 95% CI: 1.16–2.68), a high adiponectin level (HR = 2.76, 95% CI: 1.08–4.61), and multi-vessel lesions (HR = 2.67, 95% CI: 1.13–6.21) were associated with MACEs. The risk of MACEs was significantly higher in quartiles 3 (*P*=0.042) and 4 (*P*=0.0100) compared with quartile 1. Kaplan–Meier analyses indicated a significant difference in the survival rate between quartiles 1 and 4 (*P*=0.019, Figure 1) but no differences among the remaining groups. Table 3 presents pairwise comparisons among the different EATV quartiles. After adjusting for confounding factors, quartile 4 was still associated with MACEs (HR = 1.98, 95% CI: 1.15–4.47, *P*=0.023) compared with quartile 1. Other factors associated with MACEs were age ≥ 60 years, BMI ≥ 28, blood glucose level ≥ 6.1, high adiponectin, and multi-vessel involvement (Table 4). The restrictive cubic spline plots indicated a dose–response relationship between EATV and the HRs for MACEs (Figure 2). We performed another Cox regression analysis of the factors as continuous variable (age, BMI, and blood glucose level), and the results remained the same (Table 5).

**Table 2:** Univariate Cox regression analysis to assess independent correlated of adverse events following PCI for CHD during follow-up

Factors	*B*	*SE*	*Waldχ^2^*	*P*-value	HR (95% CI)
Age ≥ 60	0.841	0.021	19.854	0.000	2.32 (1.87–6.15)
Male	0.125	0.495	0.064	0.365	1.13 (1.06–2.69)
Hypertension	−0.400	0.408	0.961	0.327	0.67 (0.30–1.49)
Diabetes	−0.210	0.546	0.148	0.701	0.81 (0.28–2.36)
Smoking	0.404	0.457	0.783	0.376	1.50 (0.61–3.67)
Drinking	0.465	0.515	0.814	0.367	1.59 (0.58–4.37)
BMI ≥ 28 kg/m^2^	0.608	0.156	17.228	0.000	1.84 (1.35–2.49)
Blood glucose ≥ 6.1 mmol/l	0.044	0.018	11.612	0.013	1.04 (1.01–1.08)
TC ≥ 5.18 mmol/l	0.623	0.865	0.533	0.465	1.88 (0.34–10.25)
TG ≥ 1.7 mmol/l	−0.391	0.861	0.212	0.665	0.67 (0.12–3.64)
LDL-C ≥ 3.37 mmol/l	−0.396	0.482	0.658	0.417	0.68 (0.27–1.74)
HDL-C < 1.04 mmol/l	−0.271	0.464	0.339	0.763	0.76 (0.31–1.89)
Hs-CRP, mg/dl	0.332	0.131	13.395	0.011	1.39 (1.16–2.68)
Creatinine, μmol/l	0.124	0.110	1.722	0.125	1.13 (0.89–2.64)
Uric acid, μmol/l	0.013	0.102	0.036	0.879	1.01 (0.98–3.12)
Adiponectin, mg/l	1.015	0.478	11.007	0.018	2.76 (1.08–4.61)
Multiple vessel (*n*, %)	0.709	0.297	10.210	0.025	2.67 (1.13–6.21)
LVEF (%)	0.122	0.073	1.268	0.093	1.13 (0.98–1.30)
ACS	0.354	0.187	3.569	0.059	1.43 (0.99–2.06)
EATV					
Quartile 1					1.00
Quartile 2	0.895	0.673	8.642	0.062	2.45(0.94–3.61)
Quartile 3	1.220	0.301	10.892	0.042	3.39(1.03–3.38)
Quartile 4	1.525	0.527	11.470	0.010	4.60(1.68–6.27)

Abbreviations: ACS, acute coronary syndrome; AMI, acute myocardial infarction; BMI, body mass index; EATV, epicardial adipose tissue volume; HDL, high density lipoprotein; Hs-CRP, high-sensitivity C-reactive protein; LDL, low density lipoprotein; LEVF, left ventricular ejection fraction; LEVF; TC, total cholesterol; TG, triglyceride; UAP, unstable angina pectoris.

**Table 3:** Pairwise comparisons of survival time among different quartile of EAT

Quartile	<Q25		Q25–Q50		Q50–Q75		Q75>	
	χ^2^	*P*	χ^2^	*P*	χ^2^	*P*	χ^2^	*P*-value
<Q25	-	-	1.804	0.179	1.689	0.194	5.472	0.019
Q25–Q50	1.804	0.179	-	-	0.079	0.779	1.444	0.230
Q50–Q75	1.689	0.194	0.079	0.779	-	-	0.980	0.322
>Q75	5.472	0.019	1.444	0.230	0.980	0.322	-	-

**Table 4:** Multivariate COX regression analysis to assess independent correlated of adverse events following PCI for CHD during follow-up

Factors*	*B*	*SE*	*Waldχ^2^*	*P*-value	HR (95% CI)
Age ≥ 60	0.521	0.262	32.183	0.000	1.68 (1.29–2.11)
BMI ≥ 28 kg/m^2^	0.140	0.398	10.814	0.031	1.15 (1.09–2.36)
Blood glucose ≥ 6.1 mmol/l	0.248	0.130	10.167	0.043	1.28 (1.02–2.15)
Adiponectin, mg/l	0.593	0.471	12.301	0.006	1.81 (1.21–3.78)
Multiple vessel (*n*, %)	0.528	0.213	12.501	0.012	1.70 (1.26–3.74)
EATV					
Quartile 2	0.147	0.357	5.398	0.128	1.16 (0.71–2.49)
Quartile 3	0.045	0.174	4.891	0.130	1.05 (0.81–1.62)
Quartile 4	0.682	0.369	10.168	0.023	1.98 (1.15–4.47)

*adjusting potential confounding factors, including age, gender, smoking, drinking, hypertension, diabetes mellitus, BMI, TC, TG, LDL-C, HDL-C, hs-CRP, creatinine, uric acid, vessel lesions, type of CAD, and LVEF.

**Table 5:** Multivariate COX regression analysis to assess independent correlated of adverse events following PCI for CHD during follow-up (age, BMI, and blood glucose as continuous variables)

Factors*	*B*	*SE*	*Waldχ^2^*	*P*-value	HR (95%CI)
Age(year)	0.132	0.012	7.411	0.006	1.14 (1.11–1.56)
BMI (kg/m^2^)	0.330	0.021	9.246	0.002	1.39 (1.12–2.19)
Adiponectin, mg/L	0.223	0.044	25.491	0.000	1.25 (1.18–4.06)
Multiple vessel (n, %)	0.370	0.175	4.471	0.034	1.45 (1.03–2.04)
EATV					
Quartile 2	0.462	0.443	2.065	0.151	1.59 (0.78–4.76)
Quartile 3	0.586	0.460	1.621	0.203	1.80 (0.73–4.43)
Quartile 4	0.660	0.459	10.241	0.032	1.94 (1.10–4.76)

*adjusting potential confounding factors, including age, gender, smoking, drinking, hypertension, diabetes mellitus, BMI, TC, TG, LDL-C, HDL-C, hs-CRP, creatinine, uric acid, vessel lesions, type of CAD and LVEF.

## Discussion

The present study analyzed the relationships between EATV and risk factors for CAD, as well as the influence of EATV on the rate of MACEs in patients with CHD undergoing PCI. Comparing the event-free survival rate among the EATV quartiles, rate was significantly higher in the patients of quartile 4 compared with quartile 1. The quartile 4 EATV was a significant independent predictor of an increased MACE rate following PCI in patients with CHD, even after adjusting for several important demographic, clinical, and angiographic factors.

EAT is deposited directly around the coronary arteries and pericardium. An increased EATV is significantly associated with the formation and composition of coronary atherosclerotic plaque, degree of coronary luminal stenosis, and progression of calcification. However, few studies have evaluated whether an increased EATV can exacerbate the incidence of MACEs in patients with CHD after PCI, especially in the long-term. A recent study also reported a relationship between EATV and the clinical prognosis of patients with CHD after PCI but differed in some regards from our study [12]. First, that study did not present survival data and compared the incidence of MACEs among three different EATV groups. Second, they did not perform multivariate analysis, only univariate analyses. Nevertheless, the authors still concluded that EATV is an independent risk factor for MACEs in patients with CHD after PCI, which may be an inappropriate conclusion. Third, the data they collected were obtained within 1 year, whereas we conducted a longer follow-up. Our study also had a larger sample size and provided more support for the findings. Hauser et al. also assessed the relationship between EAT and cardiovascular outcome in patients with acute coronary syndrome undergoing PCI, using the EAT thickness measured by cardiac CT instead of EATV. However, the EAT thickness may not reflect the total quantity of EAT due to the two-dimensional nature of the measurement. Volume measurement is the most accurate way to assess the actual EAT quantity [13]. Using this method, coronary artery calcification may also be quantified, resulting in a more reliable assessment of cardiovascular risk [14].

Our results suggested that EAT is associated with a poor prognosis in patients with CHD after PCI. Some potential mechanisms might explain this relationship. It has been suggested that EAT is a risk factor for metabolic syndrome and cardiovascular disease and is anatomically very close to the adventitia of the coronary circulatory system and myocardium [15]. EAT consists of fat cells, lymphocytes, macrophages, and mast cells. EAT inflammation is involved in the development of atherosclerosis. Clinical research has confirmed the relationship between EAT and CHD, with the cellular and molecular effects of EAT on CHD becoming increasingly apparent [16]. The pathogenesis of CHD may be from “outside to inside” rather than that proposed by the traditional model, in which endothelial tissue is damaged by intravascular conditions, such as hypertension, hyperplasia, and the formation of lipid core coronary plaques [17]. Neovascularization/vasa vasorum neovascularization and vascular-remodeling influence the progression and composition of vulnerable coronary plaques [18]. During the early stages of atherosclerotic lesion development in coronary arteries, intimal thickening causes smooth muscle cells to secrete hypoxia-inducible factor (HIF). HIF and other inflammatory cytokines, such as TNF-α, can induce neointimal thickness and fatty streaks in the vasa vasorum [19]. Recent evidence suggests that EAT secrets inflammatory HIFs, which are involved in angiogenesis. Angiogenesis or neovascularization reduces the effect of hypoxia on intimal thickening during the early stages of coronary atherosclerotic lesion development [20]. However, the vasa vasorum can transfer harmful inflammatory cytokines and adipocytokines derived from EAT to the neointima fatty plaque. This can increase neointimal inflammation and plaque formation. Some studies have suggested that cytokines and adipocytokines derived from EAT increase lymphocyte, macrophage, and mast cell activities around perivascular adipose tissue [21]. Furthermore, macrophages migrating into plaques via the vasa vasorum and surrounding perivascular adipose tissue become a source of foam cells, which are involved in the pathogenesis of atherosclerosis. Macrophages are a major source of metalloproteinases, and evidence suggests that secretion of metalloproteinases in EAT is higher in patients with CHD than in those without CHD. The matrix metalloproteinase system plays a key role in the degradation of extracellular matrix components, whereas smooth muscle cell migration and the infiltration of macrophages are important in neointimal formation after vascular injury [22]. Degradation of the extracellular matrix, hemodynamic stimulation, elastolysis, and collagenolytic processes lead to the migration of smooth muscle cells, contributing to lasting structural changes in the vascular wall during the progression of cardiovascular disease [23]. There is evidence that the rate of fatty acid release by EAT is approximately twice that by pericardial and peri-renal tissue depots [24]. High levels of epicardial fatty acids can lead to accumulation of lipids in plaques. These results indicate that EATV and EAT-derived inflammatory cytokines are significantly correlated with plaque vulnerability in CHD. This may be one of the mechanisms by which EAT is involved in the prognosis of patients with CHD after PCI.

Our study found that blood glucose level was positively associated with adverse outcomes for CHD patients after PCI. These data were consistent with those of previous studies. Previous studies reported a positive association between hyperglycemia at the time of the event and subsequent mortality from acute myocardial infarction [25]. Moreover, Marfella et al performed a randomized, prospective, open label study to compare two therapeutic strategies after PCI with stent in patients ST-elevation myocardial infarction. They found peri-procedural tight glycemic control significantly increased the area of myocardial salvage accompanied with a reduction of the ischemic area and greater recovery of LV function at 6 months after stenting for STEMI patients, and tight control of peri-procedural plasma glucose levels were associated with improved myocardial salvage independently from HbA1c levels [26]. This study suggested that the acute metabolic milieu at the time of the PCI is more significant than chronic glycemic control in setting the stage for myocardial salvage, and confirmed us the importance of better glucose homeostasis to regulate regenerative processes during STEMI events in Type 2 diabetes mellitus (T2DM) patients treated by PCI.

T2DM patients usually have higher levels of advanced glycation end products, which damaged systemic arterial endothelial cell structure and function, delayed endothelial repair, and exposed the subendothelial collagen tissue. The platelet can easily adhesion on the injured arterial endothelial cell, then caused aggregation and thrombosis. The hyperglycemia also enhances the inflammatory reaction of arterioles and micro arteries, aggravates the resistance of microcirculation, causes microcirculation obstacle, and affects the blood supply and normal physiological function of myocardial cells. Increased oxidative stress and reactive oxygen species also lead to changes in vascular reactivity, plaque rupture and coronary artery spasm, and increased risk of cardiovascular events [27]. This process was involved in some key moleculars such as SIRT6. Among the SIRTs, SIRT6, a chromatin-associated deacetylase, is considered to have a leading role in regulating genomic stability, cellular metabolism, stress response, aging, and inflammation reaction. It was reported that diabetic plaques had more inflammation and oxidative stress, along with a lesser SIRT6 expression and collagen content compared with nondiabetic plaques. After taking glucagon-like peptide-1, the levels of SIRT6 increased, and inflammation and oxidative stress significantly decreased. These results were confirmed by *in vitro* experiments [28]. Therefore, the instability of atherosclerotic plaque instability in T2DM patients, and the pro-inflammatory/oxidative properties of the plaque in T2DM patients are also reflected by an excessive thrombus burden with higher dimension of thrombus during the acute coronary event and higher expression of inflammatory cells at level of thrombus in the coronary culprit lesion. An observational cohort study with 3166 patients with first STEMI assessed whether the thrombus aspiration before primary percutaneous coronary intervention may improve STEMI outcomes in hyperglycemic patients [29]. Their results indicated that thrombus aspiration was not associated with lower mortality in PCI for STEMI. Conversely, thrombus aspiration during PCI for STEMI reduces clinical outcomes in hyperglycemic patients. The present study suggested that hyperglycemic overload may affect not only the endothelial functionality and atherosclerotic plaque, but also the pro-thrombotic properties in coronary vessels, and long-term clinical outcomes and worse prognosis in affected patients. Some treatment methods may affect the prognosis of T2DM patients, admitted for STEMI. A study evaluated the 12-month prognosis of patients with multivessel non-obstructive coronary stenosis-diabetics previously treated with incretin-based therapy and never treated with such therapy. The found that never-incretin-users have worse prognosis as compared with current-incretin-users in diabetic patients with STEMI [30]. For patients with non-obstructive coronary artery stenosis (NOCS)-non-ST-elevation myocardial infarction, they found similar results [31].

AF is the most common sustained cardiac arrhythmia, which can worsen congestive heart failure and cerebrovascular accident and was considered as a major contributor of increased morbidity and mortality. There are numerous and different epigenetic, molecular, and cellular processes that might be implied in worse prognosis such as miRNAs. MiRNAs have been used as AF fibrotic and electrical alterations biomarkers [32]. MiRNAs were involved in many pathophysiological processes and were also used for diagnostic and prognosis biomarker in CHD patients such as microRNA-128, microRNA18a [33,34]. Moreover, in AF patients there is a consistent, chronic alteration/unbalance of the inflammation/oxidative stress that might be the result of an over activation of the EAT as dimension and as endocrine gland. Because the serum inflammation biomarker significantly alters anti-oxidant treatment in AF patients with catheter ablation [35]. The catheter ablation treatment may initiate an acute inflammatory response that could affect atrial function and post-ablation outcomes. Further research is needed.

Our study had several limitations. First, it was a single-center, retrospective analysis of an existing database. It is not known whether residual confounders affected the outcomes despite the use of multivariate analysis; inflammation is involved in the process of CHD, but certain inflammatory factors were not evaluated. Our findings need to be confirmed in larger, sufficiently powered, randomized studies with long-term follow-up periods.

In conclusion, EATV is associated with MACEs in patients with CHD undergoing PCI, and an EATV > 125.2 cm^3^ was an independent predictor of MACEs in these patients. Inflammation is involved in the effects of EAT on CHD. Further studies of larger cohorts are needed to confirm our findings.

## Abbreviations

AF: atrial fibrillation
BMI: body mass index
CAD: coronary artery disease
CHD: coronary heart disease
CT: computed tomography
EAT: epicardial adipose tissue
EATV: epicardial adipose tissue volume
ECG: electrocardiogram
HDL-C: high-density lipoprotein cholesterol
HIF: hypoxia-inducible factor
hs-CRP: high-sensitivity C-reactive protein
LBBB: left bundle branch block
LDL-C: low-density lipoprotein cholesterol
LVEF: left ventricular ejection fraction
MACE: major adverse cardiovascular event
PCI: percutaneous coronary intervention
T2DM: Type 2 diabetes mellitus
TC: total cholesterol
TG: triglycerides
